# Quantity over quality? Prey-field characteristics influence the foraging decisions of little penguins (*Eudyptula minor*)

**DOI:** 10.1098/rsos.211171

**Published:** 2022-06-15

**Authors:** G. J. Sutton, J. P. Y. Arnould

**Affiliations:** School of Life and Environmental Sciences, Faculty of Science and Technology, Deakin University, 221 Burwood Highway, Burwood, VIC 3125, Australia

**Keywords:** penguin, camera, patch size, prey, foraging, predator–prey interactions

## Abstract

Quantifying prey characteristics is important for understanding the foraging behaviour of predators, which ultimately influence the structure and function of entire ecosystems. However, information available on prey is often at magnitudes which cannot be used to infer the fine-scale behaviour of predators, especially so in marine environments where direct observation of predator–prey interactions is rarely possible. In the present study, animal-borne video data loggers were used to determine the influence of prey type and patch density on the foraging behaviour of the little penguin (*Eudyptula minor*), an important predator in southeastern Australia. We found that numerical density positively influenced time spent foraging at a patch. However, when accounting for calorific value in density estimates, individuals spent longer at dense patches of low-quality prey. This may reflect a trade-off between capture effort and calorific gain as lower quality prey were captured at higher rates. During the breeding season, foraging trip distance and duration is constrained by the need to return to the colony each day to feed offspring. The results of the study suggest that, under these spatio-temporal constraints, little penguins maximize foraging performance by concentrating efforts at larger quantities of prey, irrespective of their calorific quality.

## 1. Introduction

Successfully finding and capturing prey is the most important aspect of an animal's fitness as it provides the energy required to carry out important daily processes [1,2]. When prey availability changes, animals are expected to adapt their responses in order to continue maximizing their energy gain while minimizing energy expenditure [3,4]. Energy acquisition through efficient foraging strategies directly impacts an animal's fitness, especially during important life-history periods such as breeding [5]. Understanding how foraging and energy use are intertwined, especially during periods of high-energy demand, is important for predicting population trends in a changing environment [6].

Predators have the potential to place extraordinary pressures on their prey populations, which can lead to changes in the structure of ecosystems [7,8]. The strength of top-down effects in any system is influenced by the population of predators and the availability of prey [9,10]. In terrestrial environments, researchers have defined and quantified the ecological roles of many large carnivore species [11–13]. However, in marine environments, with many predator species hunting at depth, direct assessment of foraging behaviour and predator–prey interactions is logistically difficult. While there have been attempts to quantify prey presence through the use of meso-scale environmental characteristics (e.g. sea surface temperature (SST) and Chlorophyll-*a*), and at-sea sonar surveys, studies have revealed mis-matches between these variables and the distribution of predators suggesting time lags associated with these data [14]. The use of telemetry such as GPS [15], dive recorders [16], accelerometers [17,18], stomach temperature and mandibular sensors [19–22] have also been used to infer prey capture attempts. However, such devices do not provide the ability to directly quantify an animal's prey-field (i.e. the type and density of prey which is immediately available to the forager), a critical component in quantifying predator–prey interactions and gaining further insight into ecosystem functioning [23,24].

In the marine environment, pelagic prey is distributed in discrete patches at varying spatio-temporal scales [25]. In order to observe predator–prey encounters, animal-borne video data loggers are useful in quantifying predator movements in response to their associated prey-fields [23,24,26,27]. However, few studies have assessed foraging behaviour in response to prey abundance and quality (i.e. the energetic value) [28–30]. It is these direct prey-field measurements that allow insight into the foraging decisions of predators and how behaviour may adapt to changes in prey availability because of natural and anthropogenic environmental variation.

Breeding seabirds are ideal species to study predator–prey interactions as it is possible to obtain large amounts of information on the foraging behaviour of many individuals due to their regular nest attendance patterns and colonial breeding [31]. During breeding, they must forage for both self-maintenance and offspring provisioning [32]. Therefore, it is assumed that seabirds should target prey that is of a high-energy content in order to reduce foraging effort. However, little is known of the strategies individuals adopt when high-calorific prey is not available [10,33]. In addition, many breeding seabirds, particularly penguins (family: Spheniscidae), display a narrow foraging range due to regular nest attendance patterns and, thus, forage under both spatial and temporal constraints [34]. Understanding how individuals find and consume prey during these important periods is essential to predicting how they may respond to alterations in the composition of prey communities, which ultimately impacts their reproductive success and population survival.

The little penguin (*Eudyptula minor*) is an important consumer in the marine ecosystems of southeastern Australia, a highly variable region with some of the most rapidly warming waters in the world [35]. Due to its high cost of transport and small size, the little penguin has one of the most reduced foraging ranges of any penguin [36]. While some have suggested negative consequences in breeding success due to changes in pelagic prey availability [37], few studies have investigated how the immediate prey-field influences little penguin foraging decisions [38]. Understanding how individuals maximize their foraging success when faced with prey varying in quantity and calorific quality may provide insights into how anticipated changes to prey availability in this region may impact predator–prey interactions [39].

The aims of this study were to determine in little penguins the relationship between foraging effort and prey-field factors: (i) prey type and (ii) patch density. According to foraging theory [40,41], it is hypothesized that individuals will forage longer at prey patches of higher calorific density. It is predicted that animals should be able to distinguish between prey types to choose those with the greatest profitability. Therefore, penguins should adjust their foraging effort according to the calorific value of their prey [42–44].

## 2. Methods

To increase the likelihood of detecting varying types and densities of prey, the study was conducted at two breeding colonies in southeastern Australia: London Bridge (LB, 38°62′ S, 142°93′ E) and Gabo Island (GI, 37°56′ S, 149°91′ E). During the 2014–2015 (October–January) breeding period, adult penguins rearing small chicks were captured in their nest burrows and weighed in a cloth bag using a spring balance (±10 g).

Individuals were instrumented with a video data logger (Catnip Technologies Ltd, USA, 30 × 40 × 15 mm, 20 g, 400 × 400 pixels at 30 frames s^−1^) programmed to record continuously on a duty cycle of 15 min every hour, and a GPS (Mobile Action Technology, I-gotU, GT-120, 44.5 × 28.5 × 13 mm, 17 g) programmed to sample location every 2 min. The devices were attached along the dorsal midline using Tesa® tape (4651), with the camera facing forward. Together, they weighed 3% of the average little penguin body mass (1109 ± 17 g) and were less than 1% cross-sectional surface area. Individuals were recaptured in their nest burrows and the devices were removed after a single foraging trip. The morphometrics bill depth, bill length, head length and flipper length were measured using a Vernier calliper and ruler (±0.1 mm and 1 mm, respectively). Bill length was used to determine sex with males generally presenting with a larger bill depth than females [45]. Handling periods at deployment and retrieval did not exceed 10 min and measurements were completed on retrieval to minimize handling at deployment. After handling, individuals were released back into their nests to resume normal activity.

### 2.1. Data processing and statistical analyses

Unless otherwise stated, all data analyses were conducted in the R Statistical Environment (v. 4.0.2) [46]. The GPS location data were filtered to remove erroneous fixes that exceeded the maximum average horizontal travel speed of 7.2 m s^−1^ [36] and the foraging trip metrics range (km) and total duration (h) were determined using the *trip* package [47].

**Figure 1.**
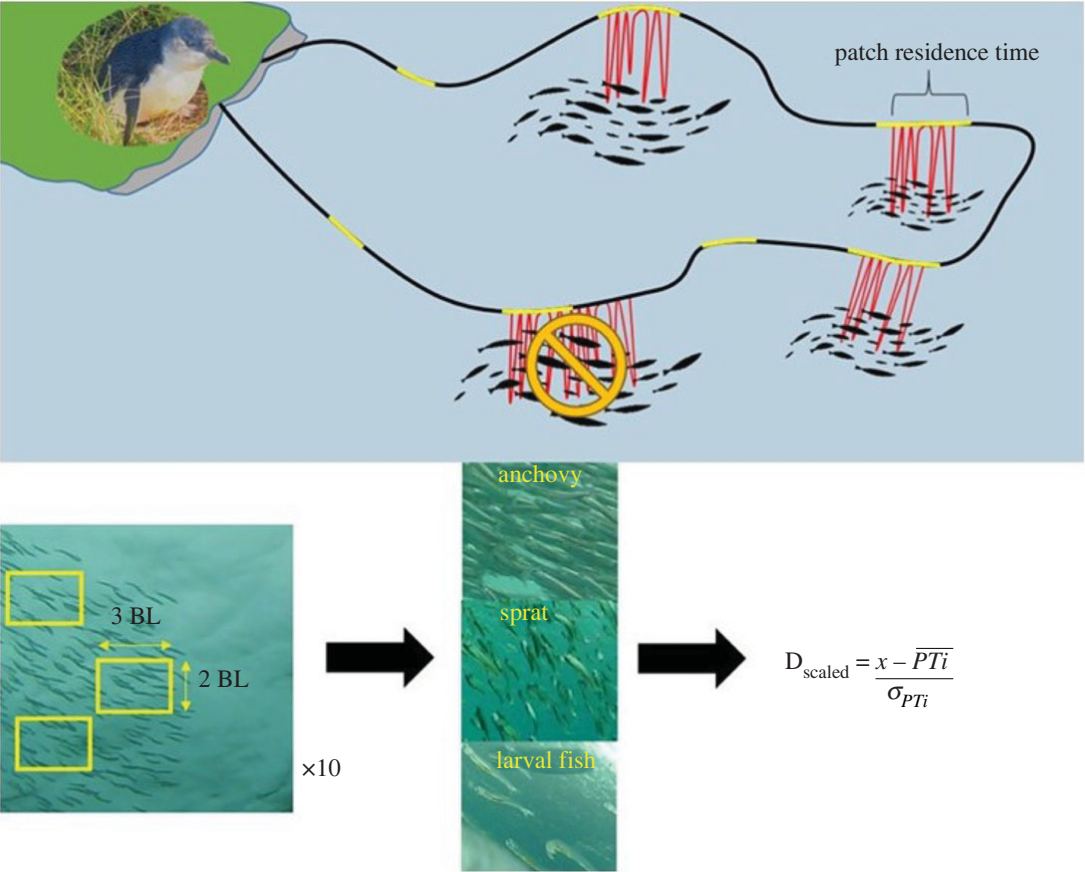
Schematic for determining prey abundance and patch residence time from animal-borne video camera data obtained from free-ranging little penguins. Little penguins were tracked with GPS (black line) and depth recorder (red lines indicating dives). Video data (yellow lines) were collected over a foraging trip at increments of 15 min every hour. Patch residence time, a proxy for foraging effort, was calculated from the beginning to the end of the first and last dive at a patch. Only prey patches that commenced and concluded during a video recording period were used. Number of fish in a window size of 3 × 2 body lengths (BL) was used as an index of abundance for each fish prey type and converted to centimetres based on average BL values. These values were subtracted from the mean density and divided by the standard deviation to obtain a scaled numerical index of patch abundance (D_scaled_).

Video data were viewed and analysed through a custom annotation sheet created in Solomon Coder (Budapest, Hungary, v. 16.06.26). Video data was used to determine prey type, prey capture events and the beginning and end of a foraging bout. For each foraging bout, prey were identified in the video data to the lowest taxonomic level possible with aid of a fish identification guide [48]. It was not always possible to determine total patch abundance as the whole prey patch was not always visible. Therefore, an index of prey patch density was calculated for each fish prey patch from a random sample of 10 still images from each foraging bout. A rectangle based on three body lengths × two body lengths of the observed fish was overlayed on the still images and all fish within that rectangle were counted. Prey items on the fringe of the rectangle were included in counts if more than 50% of their body was inside the area. This was repeated three times for each image with rectangles being placed at random on the bait ball (figure 1). The physical height and length of each polygon was then estimated from the body length of each fish species from literature [48]. Larval fish were present in video footage and, as it was not possible to determine the species, the average larval fish size for all Clupeiformes species present in this study was used [38]. These values were used to determine the numerical window size and calculate estimates of density (number of prey m^−2^) which was then standardized to control for different window sizes between prey types using the following equation:

$${\text{D}}_{\mathrm{scaled}}=\frac{x-\;\bar{PT}}{\sigma_{PT}},$$

where D_scaled_ is the scaled density value, *x* is the non-scaled value, the mean $\left(\bar{PT}\right)$ and the standard deviation (σ) for prey type density (*PT*).

As an indication of prey quality, energetic content of prey types was estimated from a previous study [38]. These gross energy (kJ) estimates were multiplied by the densities and scaled using the same above formula. These were used to provide ecological context for foraging decisions. Penguins are known to forage on prey patches through series of dives, called a dive bout [49]. Directly after a bout of foraging, individuals generally display either resting or travelling periods. Hence, a foraging bout was considered to have ceased if an individual spent greater than 1 min resting or preening on the surface or if there were consecutive shallow travelling dives and no prey were encountered. Patch residency time (PRT, min) was used as a proxy for effort and was defined as the beginning of the first dive in which the individual encountered a prey patch to the end of the last dive in a patch. Foraging bouts were removed from further analysis if it was not possible to identify the beginning and end of the event (e.g. if the video period ended before the individual ceased foraging).

Linear mixed effects (LME) models were used to determine the factors influencing PRT using the *lme4* package [50]. Initial comparisons revealed no differences in PRT between sexes or colonies and so data were pooled. The assumptions of homoscedasticity and normal distribution of residuals were tested using Levene's tests and visual inspection of qq-plots, respectively. This revealed PRT was positively skewed, which was subsequently improved through log transformation. The model included prey type, D_scaled_ and an interaction between the two variables. To determine if effort is influenced by the energetic quality of a patch, a second LME was performed which included prey type, the calorific density and an interaction between the two variables. All models included individual as a random factor to account for repeated measures. Model selection was undertaken using the *MuMIn* package [51] to determine the most parsimonious combination of predictor variables. The best model was determined as the one with the lowest Akaike information criterion, corrected for small sample sizes (AICc) and candidate models with a ΔAICc of less than 4 are presented as the likely models for explaining PRT [52]. Unless otherwise stated, data are presented as mean ± s.e.

## 3. Results

A total of 22 little penguins (11 from each colony) were instrumented for a single foraging trip, which lasted on average 15 ± 0.4 h during which individuals travelled a total of 45.06 ± 3.8 km near the colony (range: 18.5 ± 1.4 km; table 1). Ten of the 22 individuals gained between 10 and 130 g after a single foraging trip. However, as birds were captured after they had returned to their burrows, these values are an imprecise representation of foraging success as it is possible that they had already fed their chicks.

**Table 1.** Summary of camera and GPS deployments on little penguins from two colonies (Gabo Island, GI and London Bridge, LB) in southeastern Australia. The number of video intervals and associated prey patches, total captures, dives within patches and the average patch capture rate are provided.

colony	deployment date	ID	sex	deployment mass (kg)	retrieval mass (kg)	video intervals (*N*)	number of patches	total captures	total dives	average capture rate
GI	13/01/2015	LP01	F	1.1	1.08	18	70	115	86	1.6
GI	13/01/2015	LP02	F	1.01	1.04	12	6	9	14	1.5
GI	29/11/2014	LP03	M	1.16	1.23	17	9	23	30	2.6
GI	29/11/2014	LP04	M	1.24	1.21	13	3	2	3	0.67
GI	30/11/2014	LP05	F	1.11	1.19	18	32	26	32	0.81
GI	30/11/2014	LP06	F	1.12	1.25	14	14	8	14	0.57
GI	30/11/2014	LP07	M	1.14	1.24	12	5	11	18	2.2
GI	13/01/2015	LP08	M	1.12	1.09	13	31	77	59	2.5
GI	13/01/2015	LP09	M	1.03	1	12	35	82	62	2.3
GI	13/01/2015	LP10	M	1.08	1.05	11	15	18	16	1.2
GI	13/01/2015	LP11	F	1.09	1.06	12	11	40	63	3.6
LB	10/12/2014	LP12	M	1.07	1.16	11	29	31	35	1.1
LB	09/11/2014	LP13	F	1.01	1.09	15	1	4	20	4
LB	10/12/2014	LP14	F	1	1.02	18	7	21	28	3
LB	28/12/2014	LP15	M	1.17	1.11	19	5	30	31	6
LB	27/12/2014	LP16	M	1.14	1.15	15	4	5	4	1.3
LB	27/12/2014	LP17	M	1.2	1.14	12	8	8	8	1
LB	27/12/2014	LP18	F	1.07	1.05	7	14	15	14	1.1
LB	27/12/2014	LP19	F	0.94	0.99	20	6	6	6	1
LB	28/12/2014	LP20	F	1.03	1.07	17	5	14	43	2.8
LB	27/12/2014	LP21	M	1.14	1.17	16	8	57	126	7.1
LB	28/12/2014	LP22	M	1.06	1.02	19	2	18	64	9

Video data provided on average 4.7 ± 1.1 h of the foraging trip, accounting for approximately 25% of each individual trip. A total of 320 prey patches were identified, where penguins completed 776 dives (range: 1–36 dives per patch) and consumed a total of 620 prey items. Four main prey species were identified from the video data: southern anchovy, *Engraulis australis*; sandy sprat, *Hyperlophus vittatus*; juvenile Clupeiformes (hereafter referred to as larval fish) and jellyfish, *Cyanea* spp. (figure 1).

**Figure 2.**
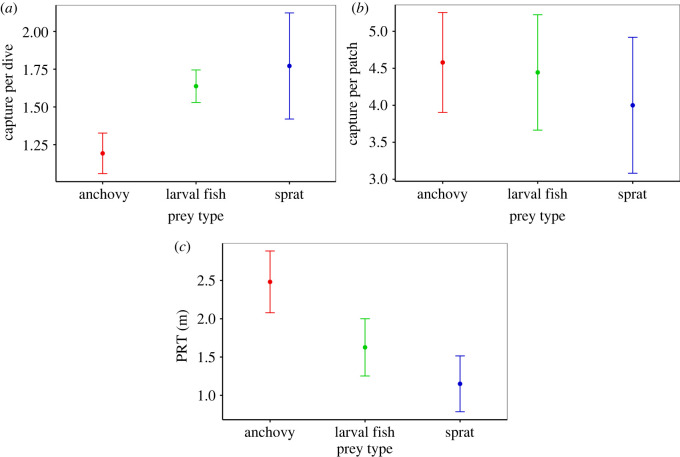
Average captures per dive (*a*), per prey patch (*b*) and PRT (*c*) for prey types (anchovy, larval fish and sprat) encountered by little penguins.

Numerical and calorific densities were the highest during encounters of anchovy, followed by sprat and larval fish (table 2). The lowest numerical and calorific densities were of jellyfish as they do not school closely together. These were removed from further analyses (*N* = 103) along with prey captures of solitary prey items (representing 29% of all prey captures). Individuals consumed on average 4.7 ± 1 fish per prey patch and spent between 0.5 and 9.6 min foraging at a single patch (mean 12.1 ± 0.2 min). These metrics seemed to be influenced by prey type as penguins spent longest at patches of anchovy, followed by larval fish and sprat (figure 2). Although prey captures per dive were highest for larval fish and sprat, there was no difference between average number of prey captured at the patch scale (figure 2). There were no instances where the prey patch was consumed entirely, with individuals ceasing foraging when the prey patch had dispersed.

**Table 2.** Summary of prey events by prey type, the proportion of prey captures which were categorized as occurring as part of a foraging bout, range of observed prey patch densities, capture rates and estimated energy content.

prey type	events (*N*)	total prey captures	patch events (%)	density (m^2^)	captures per dive ± s.e.	captures per patch ± s.e.	energy content (kJ g^−1^)
anchovy	93	241	40.4	0.04–38.4	1.2 ± 0.1	4.5 ± 0.7	5.2
sprat	29	59	34.5	2.6–25.2	1.8 ± 0.3	4 ± 0.9	5
larval fish	95	218	37.9	5.5–29.2	1.6 ± 0.1	4.4 ± 0.8	2.2

**Figure 3.**
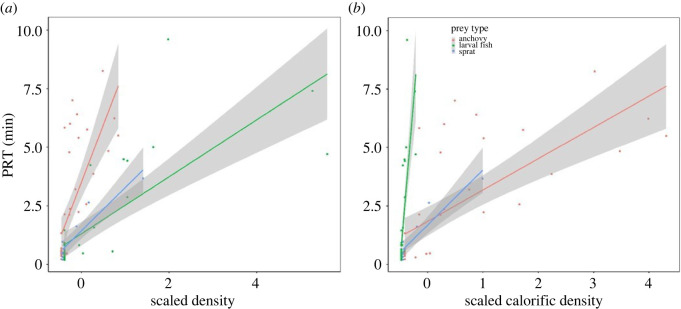
PRT (min) for scaled numerical density of each prey type (*a*) and scaled calorific density (*b*). Shaded area represents the 95% confidence interval and black line indicates overall relationship between PRT and D_scaled_.

The most parsimonious model explaining PRT included numerical patch density, prey type and an interaction between the two variables (table 3). This model accounted for more than 81% of the variation observed in PRT and was the only candidate model with a ΔAICc of less than 4. In general, penguins spent significantly longer at prey patches of higher numerical densities of prey (figure 3). However, the interaction term indicated that the relationship between numerical density and PRT was prey specific with individuals spending longer at anchovy patches than sprat and larval fish (table 3). When accounting for the calorific value in density estimates, the model followed a similar overall trend across all prey types with individuals spending longer at patches of higher calorific densities. However, the interaction term revealed that penguins spent significantly longer at densities of larval fish even though it was of lower calorific density/quality than the other prey types (figure 3).

**Table 3.** Model summary table for foraging effort (PRT) in little penguins. The predictors: numerical density (D_scaled_), PT(prey type) and calorific density (kJ D^−1^) were all retained in the most parsimonious models. Significant predictor effects (*p* < 0.05) are indicated in italics.

response	predictors	estimate	s.e.	*t*-value	CI	*p*
PRT	intercept	0.98	0.19	5.16	0.62, 1.34	*<0.001*
	D_scaled_	2.07	0.39	5.26	1.32, 2.83	*<0.001*
	PT(larval fish)	−0.84	0.22	−3.76	−1.28, −0.41	*<0.001*
	PT(sprat)	−0.69	0.34	−2.06	−1.35, −0.04	*0.04*
	D_scaled_ * PT(larval fish)	−1.61	0.40	−4.07	−2.37, −0.86	*<0.001*
	D_scaled_ * PT(sprat)	−1.05	0.54	−1.93	−2.09, 0.01	0.06
PRT	intercept	0.27	0.19	1.40	−0.10, 0.64	0.1
	kJ D^−1^	0.57	0.11	5.26	0.36, 0.77	*<0.001*
	PT(larval fish)	4.72	1.12	4.20	2.57, 6.86	*<0.001*
	PT(sprat)	0.16	0.34	0.46	−0.52, 0.81	0.6
	kJ D^−1^ * PT(larval fish)	10.10	2.46	4.1	5.41, 14.80	*<0.001*
	kJ D^−1^ * PT(sprat)	0.74	0.49	1.50	−0.20, 1.71	0.1

## 4. Discussion

Being visual predators, little penguins are constrained to forage only during daylight hours, with all individuals in the present study returning to the colony at sunset [53]. Consistent with previous studies [36,54], individuals in the present study displayed narrow foraging ranges during the breeding season, highlighting their spatial and temporal foraging constraints. Subject to these constraints, penguins maximized their capture rate over short periods by seeking to exploit patches where prey were present in high densities, irrespective of the potential calorific value.

Optimal foraging theory suggests that individuals should make decisions to forage in a way that uses the least amount of energy for the most amount of energy gained [30]. When prey is distributed throughout the environment in discrete patches, such as in the present study, individuals are expected to leave a patch when the rate of gain drops to that of the average gain within the environment [40,41]. As prey types can vary in calorific quality, effort at a single patch should change according to prey type [55]. However, individuals in the present study did not adjust their foraging decisions as expected. This may be due to the spatio-temporal constraints imposed on them during breeding.

Outside the breeding season, little penguins have been recorded spending between 2 and 49 days at sea and foraging at maximum distances of 147 km from the colony [56]. In comparison, during early chick-rearing, foraging range and duration are highly restricted, with individuals typically alternating between guarding the chick in the nest and foraging each day. In the present study, the spatio-temporal restrictions on the foraging range of penguins may have resulted in the unbiased behaviour towards all prey types. Particularly since, during early breeding, individuals were unable to travel further afield for better quality prey and still meet offspring provisioning requirements.

Although little penguins are considered to be generalist foragers [56], it was predicted that individuals should still differentiate between high- and low-quality prey types in order to maintain the necessary time-energy balance. However, in the present study, patch numerical density was an important factor in predicting effort. Increased residency time at patches of higher density may also imply that penguins do not have prior knowledge of the habitat quality. Indeed, little penguins display low foraging site fidelity, rarely overlapping in foraging locations on consecutive trips [57]. This probably reflects the highly dynamic nature of the marine environment and the mobility of pelagic prey.

When accounting for gross energy values in density estimates, individuals spent longer at patches of larval fish than anchovy or sprat. Although larval fish are of lower calorific quality, it is possible penguins may spend a greater time at these patches because fish at this life stage are less mobile in comparison with adult fish and, thus, are easier to capture [58]. Indeed, individuals at larval fish patches had a significantly higher capture rate per dive than anchovy, probably reflecting their faster handling/processing times. As larval fish had the greatest numerical densities in the present study, this may have enabled penguins to consume larger quantities of these prey before the patches dispersed. The significantly higher capture rate per dive observed when feeding on larval fish could indicate that penguins compensate for the lower calorific value of these prey items by consuming larger quantities per dive. This could ultimately result in a similar calorific gain across all prey types.

The increased time at patches of larval fish may reflect a trade-off between capture effort and energetic gain. Exerting less effort in capturing prey may result in overall higher energy gained and could also explain why penguins were observed to consume gelata [59]. Minimizing effort is important for seabirds such as penguins, where the cost of transport is higher than for flying seabirds [60,61]. In addition, individuals may stay longer at prey patches, especially if time between foraging bouts is unpredictable [62]. Such responses have also been shown in terrestrial species experiencing constraints in their foraging time [63]. In the present study, PRT was used as a metric of foraging effort which considers time, not energy use. Although time has been shown to correlate well with energy use [60], future studies should consider using accelerometers to better understand energy expenditure and gain associated with foraging on certain prey types.

The present study revealed the fine-scale foraging decisions made by little penguins in relation to their immediate prey-field. The results indicated that individuals are influenced by the density of prey at a location rather than its potential calorific value. This probably reflects the spatio-temporal constraints associated with breeding and the dynamic nature of pelagic prey in the marine environment. Individuals may choose prey patch quantity over calorific quality during their time- and distance-limited foraging trips. However, it is important to note that the foraging decisions observed in the present study may not be representative of the species' annual cycle. Indeed, several seabird species have been found to extend their range and use alternative strategies outside of the breeding season [64]. Future studies should consider investigating how the prey-field influences foraging behaviour outside of the breeding season.

## Data Availability

The data that support the findings of this study are openly available in Zenodo at https://doi.org/10.5281/zenodo.6403771 [65] and https://doi.org/10.5281/zenodo.6413352 [66]. R code and the information used to generate modelling results are available as electronic supplementary material [67].

## References

[1] Benoit-Bird K. 2004 Prey caloric value and predator energy needs: foraging predictions for wild spinner dolphins. Mar. Biol. **145**, 435-444. (10.1007/s00227-004-1339-1)

[2] Borges I, Soares AO, Magro A, Hemptinne JL. 2011 Prey availability in time and space is a driving force in life history evolution of predatory insects. Evol. Ecol. **25**, 1307. (10.1007/s10682-011-9481-y)

[3] Cooper S. 1991 Optimal hunting group size: the need for lions to defend their kills against loss to spotted hyaenas. Afr. J. Ecol. **29**, 130-136. (10.1111/j.1365-2028.1991.tb00993.x)

[4] Heithaus MR. 2004 Predator-prey interactions. Biol. Sharks Relatives **17**, 487-521. (10.1201/9780203491317.ch17)

[5] Krebs JR, Ryan JC, Charnov EL. 1974 Hunting by expectation or optimal foraging? A study of patch use by chickadees. Anim. Behav. **22**, 953-964. (10.1016/0003-3472(74)90018-9)

[6] Boyd IL. 2002 Energetics: consequences for fitness. In *Marine mammal biology:* *an evolutionary approach* (ed. AR Hoelzel), pp. 247-277. Oxford, UK: Blackwell Science Ltd.

[7] Schmitz OJ, Suttle KB. 2001 Effects of top predator species on direct and indirect interactions in a food web. Ecology **82**, 2072-2081. (10.1890/0012-9658(2001)082[2072:eotpso]2.0.co;2)

[8] Sergio F, Caro T, Brown D, Clucas B, Hunter J, Ketchum J, McHugh K, Hiraldo F. 2008 Top predators as conservation tools: ecological rationale, assumptions, and efficacy. Ann. Rev. Ecol. Evol. Syst. **39**, 1-19. (10.1146/annurev.ecolsys.39.110707.173545)

[9] Frederiksen M, Edwards M, Richardson AJ, Halliday NC, Wanless S. 2006 From plankton to top predators: bottom-up control of a marine food web across four trophic levels. J. Anim. Ecol. **75**, 1259-1268. (10.1111/j.1365-2656.2006.01148.x) 17032358

[10] McInnes AM, Ryan PG, Lacerda M, Deshayes J, Goschen WS, Pichegru L. 2017 Small pelagic fish responses to fine-scale oceanographic conditions: implications for the endangered African penguin. Mar. Ecol. Prog. Ser. **569**, 187-203. (10.3354/meps12089)

[11] Clark CW, Mangel M. 1986 The evolutionary advantages of group foraging. Theor. Popul. Biol. **30**, 45-75.

[12] Hayward MW, Somers MJ. 2009 Reintroduction of top-order predators: using science to restore one of the drivers of biodiversity. Reintrod. Top-order Predators **7**, 1. (10.1002/9781444312034.ch1)

[13] Wilson AM et al. 2018 Biomechanics of predator–prey arms race in lion, zebra, cheetah and impala. Nature **554**, 183-188. (10.1038/nature25479) 29364874

[14] Grémillet D et al. 2008 Spatial match–mismatch in the Benguela upwelling zone: should we expect chlorophyll and sea-surface temperature to predict marine predator distributions? J. Appl. Ecol. **45**, 610-621. (10.1111/j.1365-2664.2007.01447.x)

[15] Botha JA, Pistorius PA. 2018 Variability in the foraging distribution and diet of Cape gannets between the guard and post-guard phases of the breeding cycle. Front. Mar. Sci. **5**, 15.

[16] Cook TR, Hamann M, Pichegru L, Bonadonna F, Grémillet D, Ryan PG. 2012 GPS and time-depth loggers reveal underwater foraging plasticity in a flying diver, the Cape cormorant. Mar. Biol. **159**, 373-387. (10.1007/s00227-011-1815-3)

[17] Collins PM, Halsey LG, Arnould JP, Shaw PJ, Dodd S, Green JA. 2016 Energetic consequences of time-activity budgets for a breeding seabird. J. Zool. **300**, 153-162. (10.1111/jzo.12370)

[18] Elliott KH, Le Vaillant M, Kato A, Speakman JR, Ropert-Coudert Y. 2013 Accelerometry predicts daily energy expenditure in a bird with high activity levels. Biol. Lett. **9**, 20120919. 23256182 10.1098/rsbl.2012.0919 PMC3565507

[19] Grémillet DJ, Plös AL. 1994 The use of stomach temperature records for the calculation of daily food intake in cormorants. J. Exp. Biol. **189**, 105-115. (10.1242/jeb.189.1.105) 9317430

[20] Kuhn CE, Costa DP. 2006 Identifying and quantifying prey consumption using stomach temperature change in pinnipeds. J. Exp. Biol. **209**, 4524-4532. (10.1242/jeb.02530) 17079722

[21] Naito Y. 2004 Krill-eating behaviour in a chinstrap penguin (*Pygoscelis antarctica*) compared with fish-eating in magellanic penguins (*Spheniscus magellanicus*): a pilot study. Mar. Ornithol. **32**, 754.

[22] Naito Y. 2007 How can we observe the underwater feeding behavior of endotherms? Polar Sci. **1**, 101-111.

[23] Sutton G, Pichegru L, Botha JA, Kouzani AZ, Adams S, Bost CA, Arnould JP. 2020 Multi-predator assemblages, dive type, bathymetry and sex influence foraging success and efficiency in African penguins. PeerJ **8**, e9380. (10.7717/peerj.9380) 32655991 PMC7333648

[24] Takahashi A, Kokubun N, Mori Y, Shin HC. 2008 Krill-feeding behaviour of gentoo penguins as shown by animal-borne camera loggers. Polar Biol. **31**, 1291-1294. (10.1007/s00300-008-0502-4)

[25] Weimerskirch H, Doncaster CP, Cuenot-Chaillet F. 1994 Pelagic seabirds and the marine environment: foraging patterns of wandering albatrosses in relation to prey availability and distribution. Proc. R. Soc. Lond. Ser. B **255**, 91-97. (10.1098/rspb.1994.0013)

[26] Takahashi A, Sato K, Naito Y, Dunn M, Trathan P, Croxall J. 2004 Penguin–mounted cameras glimpse underwater group behaviour. Proc. R. Soc. Lond. B **271**(Suppl. 5), S281-S2S2. (10.1098/rsbl.2004.0182) PMC1810073 15503994

[27] Thiebault A, Charrier I, Aubin T, Green DB, Pistorius PA. 2019 First evidence of underwater vocalisations in hunting penguins. PeerJ **7**, e8240. (10.7717/peerj.8240) 31976165 PMC6966993

[28] Hazen EL, Friedlaender AS, Goldbogen JA. 2015 Blue whales (*Balaenoptera musculus*) optimize foraging efficiency by balancing oxygen use and energy gain as a function of prey density. Sci. Adv. **1**, e1500469. (10.1126/sciadv.1500469) 26601290 PMC4646804

[29] Akiyama Y, Akamatsu T, Rasmussen MH, Iversen MR, Iwata T, Goto Y, Aoki K, Sato K. 2019 Leave or stay? Video-logger revealed foraging efficiency of humpback whales under temporal change in prey density. PLoS ONE **14**, e0211138. (10.1371/journal.pone.0211138) 30721236 PMC6363283

[30] Watanabe YY, Ito M, Takahashi A. 2014 Testing optimal foraging theory in a penguin-krill system. Proc. Biol. Sci. **281**, 20132376. (10.1098/rspb.2013.2376) 24478293 PMC3924065

[31] Piatt JF, Harding AM, Shultz M, Speckman SG, Van Pelt TI, Drew GS, Kettle AB. 2007 Seabirds as indicators of marine food supplies: Cairns revisited. Mar. Ecol. Prog. Ser. **352**, 221-234. (10.3354/meps07078)

[32] Boyd C, Punt AE, Weimerskirch H, Bertrand S. 2014 Movement models provide insights into variation in the foraging effort of central place foragers. Ecol. Model. **286**, 13-25. (10.1016/j.ecolmodel.2014.03.015)

[33] Montevecchi W, Benvenuti S, Garthe S, Davoren G, Fifield D. 2009 Flexible foraging tactics by a large opportunistic seabird preying on forage- and large pelagic fishes. Mar. Ecol. Prog. Ser. **385**, 295-306. (10.3354/meps08006)

[34] Croxall J, Davis L. 1999 Penguins: paradoxes and patterns. Mar. Ornithol. **27**, 1-12.

[35] Lough JM, Hobday AJ. 2011 Observed climate change in Australian marine and freshwater environments. Mar. Freshw. Res. **62**, 984-999. (10.1071/mf10272)

[36] Hoskins AJ, Dann P, Ropert-Coudert Y, Kato A, Chiaradia A, Costa DP, Arnould JP. 2008 Foraging behaviour and habitat selection of the little penguin *Eudyptula minor* during early chick rearing in Bass Strait, Australia. Mar. Ecol. Prog. Ser. **366**, 293-303. (10.3354/meps07507)

[37] Dann P, Norman FI, Cullen J, Neira FJ, Chiaradia A. 2000 Mortality and breeding failure of little penguins, *Eudyptula minor*, in Victoria, 1995–96, following a widespread mortality of pilchard, *Sardinops sagax*. Mar. Freshw. Res. **51**, 355-362. (10.1071/mf99114)

[38] Sutton GJ, Hoskins AJ, Arnould JP. 2015 Benefits of group foraging depend on prey type in a small marine predator, the little penguin. PLoS ONE **10**, e0144297. (10.1371/journal.pone.0144297) 26674073 PMC4682954

[39] Ropert-Coudert Y et al. 2019 Happy feet in a hostile world? The future of penguins depends on proactive management of current and expected threats. Front. Mar. Sci. **6**, 248. (10.3389/fmars.2019.00248)

[40] MacArthur RH, Pianka ER. 1966 On optimal use of a patchy environment. Am. Nat. **100**, 603-609. (10.1086/282454)

[41] McNair JN. 1982 Optimal giving-up times and the marginal value theorem. Am. Nat. **119**, 511-529. (10.1086/283929)

[42] Patenaude-Monette M, Bélisle M, Giroux JF. 2014 Balancing energy budget in a central-place forager: which habitat to select in a heterogeneous environment? PLoS ONE **9**, e102162. (10.1371/journal.pone.0102162) 25029498 PMC4100874

[43] Stephen D, Krebs J. 1986 Foraging behaviour. Princeton, NJ: Princeton University of Press.

[44] Thum M. 1989 The significance of opportunistic predators for the sympatric carnivorous plant species, *Drosera intermedia* and *Drosera rotundifolia*. Oecologia **81**, 397-400. (10.1007/bf00377090) 28311195

[45] Arnould JP, Dann P, Cullen J. 2004 Determining the sex of little penguins (*Eudyptula minor*) in northern Bass Strait using morphometric measurements. Emu **104**, 261-265. (10.1071/mu04035)

[46] R Core Team. 2018 R: a language and environment for statistical computing. Vienna, Austria: R Foundation for Statistical Computing.

[47] Sumner MD, Luque S, Fischbach A, Hengl T. 2020 Package ‘trip’. See https://mran.revolutionanalytics.com/snapshot/2020-04-25/web/packages/trip/trip.pdf.

[48] Gomon MF, Glover JC, Kuiter RH. 1994 Fishes of Australia's south coast. Adelaide, Australia.

[49] Berlincourt M, Arnould JP. 2014 At-sea associations in foraging little penguins. PLoS ONE **9**, e105065. (10.1371/journal.pone.0105065) 25119718 PMC4132066

[50] Bates DM, Mächler M, Bolker B, Walker S. 2014 Fitting linear mixed-effects models using lme4. J. Stat. Softw. **67**, 1-48. (10.18637/jss.v067.i01)

[51] Barton K, Barton M. 2015 Package ‘MuMin’. Version 1.9. 18. Vienna, Austria: R Foundation for Statistical Computing.

[52] Brewer MJ, Butler A, Cooksley SL. 2016 The relative performance of AIC, AICC and BIC in the presence of unobserved heterogeneity. Methods Ecol. Evol. **7**, 679-692. (10.1111/2041-210x.12541)

[53] Klomp N, Wooller R. 1991 Patterns of arrival and departure by breeding little penguins at Penguin Island, Western Australia. Emu **91**, 32-35. (10.1071/mu9910032)

[54] Berlincourt M, Arnould JP. 2015 Influence of environmental conditions on foraging behaviour and its consequences on reproductive performance in little penguins. Mar. Biol. **162**, 1485-1501. (10.1007/s00227-015-2685-x)

[55] Werner EE, Mittelbach GG. 1981 Optimal foraging: field tests of diet choice and habitat switching. Am. Zool. **21**, 813-829. (10.1093/icb/21.4.813)

[56] Cavallo C, Chiaradia A, Deagle BE, Hays GC, Jarman S, McInnes JC, Ropert-Coudert Y, Sánchez S, Reina RD. 2020 Quantifying prey availability using the foraging plasticity of a marine predator, the little penguin. Funct. Ecol. **34**, 1626-1639. (10.1111/1365-2435.13605)

[57] Camprasse EC, Sutton GJ, Berlincourt M, Arnould JP. 2017 Changing with the times: little penguins exhibit flexibility in foraging behaviour and low behavioural consistency. Mar. Biol. **164**, 169. (10.1007/s00227-017-3193-y)

[58] Hunter JR. 1972 Swimming and feeding behavior of larval anchovy *Engraulis mordax*. Fish Bull. **70**, 821-838.

[59] Thiebot JB et al. 2017 Jellyfish and other gelata as food for four penguin species – insights from predator-borne videos. Front. Ecol. Environ. **15**, 437-441. (10.1002/fee.1529)

[60] Sutton GJ, Botha JA, Speakman JR, Arnould JPY. 2021 Validating accelerometry-derived proxies of energy expenditure using the doubly labelled water method in the smallest penguin species. Biol. Open **10**, bio055475. (10.1242/bio.055475) 33722801 PMC8034874

[61] Ellis HI. 1984 Energetics of free-ranging seabirds. In Seabird energetics (eds GC Whittow, H Rahn), pp. 203-234. Boston, MA: Springer.

[62] Davidson JD, El Hady A. 2019 Foraging as an evidence accumulation process. PLoS Comput. Biol. **15**, e1007060. (10.1371/journal.pcbi.1007060) 31339878 PMC6682163

[63] Nonacs P. 2001 State dependent behavior and the marginal value theorem. Behav. Ecol. **12**, 71-83. (10.1093/oxfordjournals.beheco.a000381)

[64] Pistorius PA, Hindell MA, Tremblay Y, Rishworth GM. 2015 Weathering a dynamic seascape: influences of wind and rain on a seabird's year-round activity budgets. PLoS ONE **10**, e0142623. (10.1371/journal.pone.0142623) 26581108 PMC4651498

[65] Sutton GJ, Arnould JPY. 2022 Quantity over quality? Prey-field characteristics influence the foraging decisions of little penguins (*Eudyptula minor*). Zenodo. (10.5281/zenodo.6403771) PMC9198507 35719883

[66] Sutton GJ, Arnould JPY. 2022 Quantity over quality? Prey-field characteristics influence the foraging decisions of little penguins (*Eudyptula minor*). Zenodo. (10.5281/zenodo.6413352) PMC9198507 35719883

[67] Sutton GJ, Arnould JPY. 2022 Quantity over quality? Prey-field characteristics influence the foraging decisions of little penguins (*Eudyptula minor*). Figshare. (10.6084/m9.figshare.c.6016972) PMC9198507 35719883

